# Chronic Pain and Selective Attention to Pain Arousing Daily Activity Pictures: Evidence From an Eye Tracking Study

**DOI:** 10.29252/nirp.bcn.8.6.467

**Published:** 2017

**Authors:** Masoumeh Mahmoodi-Aghdam, Mohsen Dehghani, Mehrnoosh Ahmadi, Anahita Khorrami Banaraki, Ali Khatibi

**Affiliations:** 1. Department of Cognitive Psychology, Institute for Cognitive Science Studies (ICSS), Tehran, Iran.; 2. Family Research Institute, Shahid Beheshti University, Tehran, Iran.; 3. Department of Psychiatry, Roozbeh Psychiatry Hospital, Tehran University of Medical Sciences, Tehran, Iran.; 4. Department of Psychology, Faculty of Economics, Administrative, and Social Sciences, Bilkent University, Ankara, Turkey.; 5. Interdisciplinary Program in Neuroscience, Bilkent University, Ankara, Turkey.; 6. National Magnetic Resonance Research Center (UMRAM), Bilkent University, Ankara, Turkey.

**Keywords:** Attentional bias, Chronic pain, Eye movement, PHODA

## Abstract

**Introduction:**

According to the pain research literature, attentional bias for pain is the mechanism responsible for the development and maintenance of fear of pain in patients with chronic pain. However, there is still some debate about the exact mechanism and the role of faster engagement versus difficulty in disengagement in the development of attentional bias.

**Methods:**

To investigate attentional bias in patients with chronic pain, we used an eye-tracker with the pictures of pain-provoking activities and compared the results with an age- and gender-matched group of pain-free participants. In addition, other measures of pain-related cognition and pain severity ratings were included to assess their contribution to the attentional bias toward pain-related information.

**Results:**

Calculating the frequency of the first fixations showed that both groups fixated initially on pain-provoking pictures compared to neutral one. Calculating the speed of fixations showed that control participants were faster in fixating on neutral stimuli, but patients with pain were faster in fixating on pain-provoking pictures, indicating a relative vigilance for the pain-related stimuli among them. These patients reported that the intensity of pain in the previous week was positively correlated with the speed of their fixation on the painful stimuli.

**Conclusion:**

Although these results did not provide unequivocal support for the vigilance-avoidance hypothesis, they are generally consistent with the results of studies using eye tracking technology. Furthermore, our findings put a question over characterization of attentional biases in patients with chronic pain by simply relating that to difficulty in disengaging from pain-related stimuli.

## 1. Introduction

According to the fear-avoidance model of chronic pain, fear of pain has a key role in the development and maintenance of persistent pain (Vlaeyen & Linton, 2012). Development of pain-related fear following an injury presumably contributes to increase in hypervigilance for somatosensory cues related to pain (Asmundson, Norton, & Vlaeyen, 2004; Asmundson, Vlaeyen, & Crombez, 2004). Hypervigilance can, in turn, result in avoidance of fear-provoking activities, subsequently increasing the likelihood of depression, disability, and persistent pain (Vlaeyen & Linton, 2012).

Recent meta-analyses have presented evidence in support of attentional bias for pain-related information among patients with chronic pain (Crombez, Van Ryckeghem, Eccleston, & Van Damme, 2013; Schoth, Nunes, & Liossi, 2012). These studies used three major paradigms: modified stroop task (Andersson & Haldrup, 2003; Asmundson, Wright, & Hadjistavropoulos, 2005; Roelofs, Peters, Zeegers, & Vlaeyen, 2002), dot-probe task (Haggman, Sharpe, Nicholas, & Refshauge, 2010; Khatibi, Dehghani, Sharpe, Asmundson, & Pouretemad, 2009; Mohammadi et al., 2012), and spatial-cueing task (Van Damme, Crombez, & Eccleston, 2004; Van Ryckeghem et al., 2013). Although these tasks are widely used, they cannot distinguish between faster engagement to the target stimulus and difficulty in disengaging from it (Schoth et al., 2012).

Eye-trackers record eye movements during the stimuli and thereby more accurately assess the pattern of attentional processing during an experimental task (Mogg, Millar, & Bradley, 2000). Yang and colleagues (2013) found that high levels of pain-related fear in patients with chronic pain was associated with attention towards health catastrophe words and a pattern of subsequent avoidance, characterized by faster disengagement and subsequent re-engagement. Similarly, Liossi et al. (2014) found evidence of engagement biases for faces with painful expressions in comparison to both neutral and other emotional faces in patients with chronic headache. Although they did not find a significant effect for subsequent avoidance, a trend was seen in this direction.

Importantly, these studies used pain-related words (Yang, Jackson, & Chen, 2013) and painful face stimuli (Liossi, et al., 2013). However, the fear of (re)injury model emphasizes hypervigilance to bodily sensations associated with pain-provoking tasks, which are subsequently be avoided due to fear of injury. Only one study used photographs of daily activities as stimuli. Dear et al. (2011) found that patients with chronic pain showed a specific bias toward movements that idiosyncratically rated them as potentially painful (Dear, et al., 2011). However, this study did not mention that this reaction was due to facilitated engagement toward those pictures or difficulty in disengagement.

In the current study, we aimed to record eyes behavior of patients with chronic pain to investigate their attentional bias toward pictures of pain arousing daily activities. We hypothesized that patients with chronic pain show an engagement bias (i.e. initial orientation of attention to pain-provoking activity pictures than neutral ones). With regard to sustained attention, two competing hypotheses could be made. One could hypothesize that patients with chronic pain, relative to healthy controls, have difficulty in disengaging from pictures of pain-provoking activities. In contrast, on the basis of the findings of Yang et al. (2013), it could be hypothesized that patients with chronic pain disengage from pictures of pain-provoking activities faster than healthy controls.

## 2. Methods

### 2.1. Participants

Twenty-two patients with chronic low back pain (n=12) or chronic lower limb pain (n=6) or both (n=4) (age range=28-54 y; Mean=38.30 y, SD=8.13 y) were invited to participate in the study. They were recruited from physiotherapy and orthopedic centers of two hospitals and all of them agreed to participate. Patients had documented evidence of pain for more than 6 months to meet IASP criteria for chronic pain. None of them had a history of major trauma or diagnosed with any neurological disorder at the time of testing. Data from two female participants were excluded due to loss of more than 25% of the trials according to the defined fixation criteria (explained in “data preparation” section) in the primary analyses. Finally, a sample of 20 (11 females) patients remained. Pain duration was between 7 months and 10 years (Mean=6.37 y; SD=5.26 y).

A nonclinical group of 20 pain-free individuals, (age range=22–55 y; Mean=35.83 y, SD=7.80 y), matched with the experimental group on age and educational level, participated in the study as the control group. Two individuals were excluded for the same reason as the group with Chronic Pain (CP) and a final sample of 18 persons (13 females) remained. Any history of traumatic injury, serious mental illnesses (such as psychosis), and existing uncorrected visual impairments were considered as exclusion criteria. Since we were using a head-mounted eye-tracker, cervical pain was another exclusion criterion. The study protocol was approved by the Ethics Committee of Institute for Cognitive Science Studies (ICSS) and all participants gave written informed consent.

### 2.2. Questionnaire measures

#### 2.2.1 Pain severity: Visual Analogue Scale (VAS)

The Visual Analogue Scale (VAS) is a 10 cm ungraded line with an anchor on the left indicating “no pain at all” and another on the right denoting “the worst intolerable pain.” Three assessments of pain severity during the previous week, the current week, and expected pain in the next week were administered.

#### 2.2.2 Depression, Anxiety and Stress Scale

The short form of Depression, Anxiety and Stress Scale (DASS with 21 items) was used to measure depression, anxiety, and stress, (7 items for each subscale). The reliability and validity of both English and Persian versions of DASS are well established (Bayani, 2010; Khatibi, et al., 2009). The Cronbach α value for each subscale in the English version have been reported as follows: anxiety=0.84, depression=0.91, and stress=0.90 (Lovibond & Lovibond, 1996). In our study, the α values were 0.86, 0.92, and 0.84 for anxiety, depression, and stress, respectively.

#### 2.2.3 Roland and Morris Disability Questionnaire (RDQ)

Roland and Morris Disability Questionnaire (RDQ) is a 24-item checklist which assesses disability level in doing daily chores. RDQ has shown good psychometric properties in different studies (Roland & Fairbank, 2000). In the current study, we used a modified version of RDQ in which the phrase “my back pain” was changed to “my pain” and its Persian version, which has been used in our study, has been proven to be a valid and reliable measure (Asghari & Nicholas, 2001; Mohammadi, Dehghani, Khatibi, Sanderman, & Hagedorn, 2015; Akbari, Dehghani, Khatibi & Vervoort, 2016). In our study, the Cronbach α was 0.76.

#### 2.2.4 Tampa Scale of Kinesiophobia (TSK)

Tampa Scale of Kinesiophobia (TSK) consists of 17 items rated on a 4-point Likert-type scale (1=extremely disagree, 4=extremely agree) to measure the fear of movement and re-injury. It has been validated and shown to have a good reliability, with Cronbach α of 0.7 to 0.8 in different samples of patients with pain (Roelofs et al., 2004; Swinkels-Meewisse, Swinkels, Verbeek, Vlaeyen, & Oostendorp, 2003). The Persian version has been used in previous studies and proven to be a valid and reliable measure (Khatibi, et al., 2009). In the current sample, the Cronbach α was 0.68.

#### 2.2.5 Pain Vigilance and Awareness Questionnaire (PVAQ)

The Pain Vigilance and Awareness questionnaire (PVAQ) is a measure of pain vigilance, comprising 16 items (e.g., I am so sensitive to pain) rated on a 6-point Likert-type scale (0=never, 5=always) with a Cronbach α value of 0.83 and a total score ranging from 0 to 90 (Roelofs, Peters, McCracken, & Vlaeyen, 2003). The Persian version has been used in previous studies and proven to be a valid measure (Khatibi, et al., 2009). In the current study, the Cronbach α value was 0.87.

### 2.3. Eye tracking task

#### 2.3.1 Stimuli

A set of 40 PHODA photographs (including 8 possible movements: lifting, bending, turning, reaching, falling, intermittent load, unexpected movement, and sustained load while standing up or sitting down with limited dynamics) and 100 pictures from IAPS (Lang, Bradley, & Cuthbert, 2008) were collected. To make sure that these images were judged consistently, especially due to probable cultural differences, 34 patients with chronic musculoskeletal pain and 20 pain-free individuals were invited to take part in a pilot study. A total of 140 images were presented individually and participants were instructed to rate the severity of pain associated with the depicted action on a VAS anchored from “not painful at all” to “the worst imaginable pain.” Picture presentation was managed by Microsoft Power point 2007 and ratings were completed on paper.

Independent t tests were performed for the painful pictures. The photographs from PHODA depicting activities which were rated significantly more painful by CP patients compared to the control subjects (P≤0.01) (the data for pilot are presented in Appendix ). IAPS pictures in which more than 70% of pilot participants (both normal and CP patients) reported as “not painful at all” were selected as neutral pictures. Accordingly, 40 neutral images qualified for final step, of them 15 images (pictures: 2026pictures: 2038, 2102, 2272, 2514, 2515, 2850, 2880, 5836, 7026, 7150, 7217, 7235, 7493, 8510) were matched with 15 PHODA images with the most comparability on complexity, presence/absence of human, outside and inside scene, luminance and resolution. This resulted in 15 painful-neutral pairs. Ten other IAPS neutral pictures (pictures: 5531, 5534, 5731, 5779, 7004, 7009, 7010, 7050, 7092, 7509) were matched with each other in valence and arousal to make 5 neutral-neutral pairs. At the end 20 pairs were compiled, of which 15 were the critical trials.

**Figure 1.**
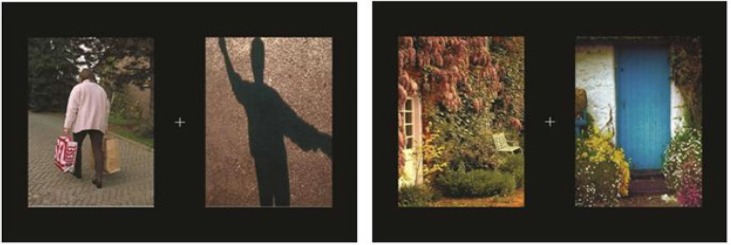
Two samples of study slides. (a): Painful-neutral; (b): Neutral-neutral

The properties of the IAPS images were slightly adjusted with Adobe Photoshop program to achieve a uniform value in the overall luminance levels, color saturation, complexity and resolution of the PHODA pictures. The intensity and contrast of IAPS pictures were also adjusted to match with PHODA images. Pictures were in JPEG format and were resized to 335×497 pixel. Experiment Builder 1.6.121 (SR Research Ltd, Mississauga, Ontario, Canada) was used to design the task. In each trial, the pictures were presented on the right or left hand side of the fixation point (Figure 1). The right image was located at 745×383 pixel and the left was located at 273×383 pixel. The innermost edges of images were 3 cm distant from the fixation point. During the task, the 20 image pairs were counterbalanced, to create 40 trials in one block. Two blocks were presented, so that the second block was identical to the first. Hence, 80 image pairs in total were presented.

#### 2.3.2 Apparatus

A 19″ AOC monitor with 1440×900 pixel screen resolution connected to a 2.60 GHz Pentium Dual core CPU computer was used to present the stimuli. Furthermore, the Eye Link II tracker (SR Research Ltd) connected to another computer with the same features was used to record the participants’ eye movements. This is a head-mounted device which uses infrared to record corneal reflection and pupil size changes. A fixation was defined as an eye position remaining within a 50 pixel area for more than 100 ms (Dyer, Found, & Rogers, 2006). The eye tracker’s sampling rate was set to 250 Hz. To follow the participants’ gaze, the right eye was used.

### 2.4. Procedure

The participants were tested individually in a quiet soundproofed room with dim light. Before the session, exclusion criteria for each participant were checked. At the beginning of the session, they received instructions regarding the experiment and signed the consent form. Then, they were asked to sit in front of the monitor at a 55 cm viewing distance. Their chins were placed in the chin-rests to prevent excessive head-movements and to ensure a constant distance from the monitor. The experimenter instructed the participant to fixate on the fixation-cross at the beginning of the task and to look freely at the pictures presented. This instruction also appeared on the monitor. Prior to the experimental task, a 9-point calibration was done to ensure that the participant’s eye movements were captured.

A white centrally located fixation cross on a black background was shown at the beginning of the task and remained constant during picture presentation. Fixation on the central cross prompted initiation of the trial. For each trial, a pair of images was displayed on a black background for 1000 ms. The fixation cross was shown again when the images disappeared. This process was repeated for the remaining trials. Once all the stimuli had been presented in each of the two possible combinations (left and right of the fixation point), a second identical block was presented. The task took approximately 5 min. After completion of the eye-tracking procedure, participants were asked to complete the battery of questionnaires. All participants completed DASS but only the pain group completed the remainder of the questionnaires.

### 2.5. Data preparation

Data collection was done using Eyelink Dataviewer. Fixations were defined as: 1. Eye movements which fell within the designated area of interest (i.e. within the boundaries of one of the pictures), 2. Occurred at least 100 ms after picture pair onset and before picture offset, and 3. Continued for at least 100 ms. Frequency and latency of first fixations were selected as indices of initial attentional bias (Vervoort, Trost, Prkachin, & Mueller, 2013; Yang, et al., 2013; Yang, Jackson, Gao, & Chen, 2012). That is, frequency and latency of first fixations assessed the degree to which the painful stimuli initially engaged participant’s attention. Frequency of total fixations, duration of the first and total fixations, as well as first run dwell time (defined as the duration of all the fixations within an interest area until the first time the participant looks outside the interest area) were also analyzed as an indication of sustained attention (Liossi, et al., 2014; Yang, et al., 2013; Yang, et al., 2012). That is, difficulties in disengaging are displayed by more and longer fixations on the pain stimuli, whereas avoidance is shown by fewer and shorter total fixations.

### 2.6. Statistical analysis

Based on the results reported by Yang et al. (2013), we anticipated a large effect size. This study was powered to find an effect size (Cohen’s d≥0.8) with a power of 80% and a significance level set at 0.05.

In preliminary analyses, we conducted a series of t tests to determine whether the order of presentation (on the left or right hand side) affected attention. The order of presentation of the slides did not make significant differences in any of the eye tracking measures as seen in the neutral-neutral condition (all t(36)≤|1.53|, ns) and therefore was not further investigated. Repeated-measures ANOVA with picture type (2: neutral vs. painful) as the within-subjects factor and group (2: chronic pain vs. control) as the between group factor were used to evaluate subjects’ selective attention toward painful images. In addition, the Pearson product-moment correlations were conducted to investigate the relationship between self-reported individual differences and indices of attentional bias.

## 3. Results

### 3.1. Demographic and Questionnaire Data

There were no differences between the groups (Chi squared=1.2, df=1, 2-tailed P=0.27), based on sex, age, and educational level. Depression score did not differ significantly between two groups. However, the average stress (t(36)=2.94, P<0.01) and anxiety (t(36)=−2.28, P<0.05) scores in patients with chronic pain were higher than healthy controls (Table 1). Given that stress and anxiety were not correlated with the indices of attention, the need to control for these variables did not arise. Nonetheless, we re-ran the analyses with these variables as covariates and the pattern of results did not changed. Therefore, we report the results of the ANOVA in Table 2.

**Table 1.** Descriptive statistics of demographic and pain-related variables

**Variables**	**Mean (SD)**		**t (36)**	**P**

	**CP (n=20)**	**Control (n=18)**		
Age (y)	38.30(8.13)	35.83(7.80)	0.95	0.34
Education level (y)	16.25(4.20)	18.27(3.99)	1.52	0.13
RDQ	7.70(3.64)	-	-	-
TSK	40.60(5.77)	-	-	-
PVAQ	46.65(13.15)	-	-	-
**DASS**				
Anxiety	4(4.63)	1.44(1.78)	2.28	0.03
Stress	8.05(4.71)	4.11(3.30)	2.94	0.006
Depression	4.3(4.86)	2.72(3.98)	1.08	0.28
**VAS**				
Past week	49.55(27.23)	-	-	-
Current week	33.85(25.60)	-	-	-
Next week	37.25(23.52)	-	-	-

DASS: Depression, Anxiety and Stress Scale; VAS: Visual Analogue Scale; RDQ: Roland and Morris Disability Questionnaire; TSK: Tampa Scale of Kinesiophobia; PVAQ: Pain vigilance and Awareness Questionnaire; CP: Chronic Pain

**Table 2.** Comparison of number of the first fixations, latency of the first fixation, number and duration of total fixations and first run dwell time for the chronic pain and control group

**Variable**	**Theme**	**Mean (SD)**	

		**CP (n=20)**	**Control (n=18)**
Number of the first fixations	Painful	27.10(6.10)	24(7.43)
	Neutral	19.15(6.04)	15.50(5.79)
Latency of the first fixations	Painful	642.59(30.71)	635.78(51.05)
	Neutral	647.18(31.89)^*^	616.52(58.00)^*^
Number of total fixations	Painful	72.80(12.87)	66.16(18.62)
	Neutral	50.15(11.37)	45.11(9.26)
Duration of first fixations	Painful	233.52(27.47)	242.84(45.41)
	Neutral	228.61(27.44)	253.94(35.05)
Duration of total fixations	Painful	222.21(21.96)	225.89(31.84)
	Neutral	225.08(21.85)	233.39(24.41)
First run dwell time	Painful	224.70(21.59)	229.81(32.08)
	Neutral	229.60(24.43)	241.90(24.26)

* P<0.05

### 3.2. Eye tracking task

#### 3.2.1 Early attentional processes

We conducted a mixed model 2 (stimuli: pain vs. neutral)×2 (group: pain vs. control) ANOVA for the number of first fixations. There was a significant main effect of stimuli type for the frequency of first fixations [F_1,36_=36.31, P<0.0001, ηp^2^=0.5], favoring the painful images (painful: Mean=25.63, SD=6.85; neutral: Mean=17.42, SD=6.13). The group×picture type interaction was not significant (F_1,36_=0.04, P=0.84). However, there was a main effect of group, indicating that overall the CP patients made more first fixations (F_1,36_=4,731, P=0.036).

**Figure 2.**
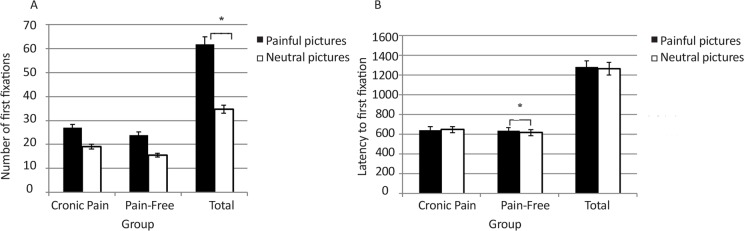
Comparison of indices of early attentional process on neutral and painful pictures between the chronic pain and pain-free groups. Generally, the number of first fixations on painful images was significantly more than that on neutral images (a significant main effect of stimuli type in mixed model ANOVA) (A). There was a significant interaction effect for latency to first fixation indicated that the control group fixated quickly on neutral images than pain images (B)

For the latency to first fixation, there was no main effect for stimuli (F_1,36_=1.803, P=0.188) or group (F_1,36_=2.021, P=0.164). However, there was a significant interaction effect (F_1,36_=4.762, P=0.036, ηp^2^=0.11), indicating that the control group fixated more quickly on neutral targets than pain targets [t(17)=2.582, P=0.019], While the CP group did not show significant differences [t(19)=0.581, P=0.57], the CP group fixated relatively quickly on pain stimuli than neutral stimuli (pain stimuli=642.59, neutral stimuli=647.18). Although the interaction effect was significant, as predicted, the groups did not differ on the speed with which the two groups fixated on the pain-related stimuli [t(36)=0.504, P=0.617] (Figure 2).

There was a significant negative correlation between VAS in the current week and first fixation latencies on painful depicting images (r=−0.569, P=0.009). This correlation indicated that participants who reported more severe pain in current week were faster to fixate on painful pictures. No other correlations were significant (P≥0.054).

#### 3.2.2 Sustained attention

For the total number of fixations, there was a significant main effect of picture type (F_1,36_=69.47, P<0.0001, ηp^2^=0.65) with most of fixations occurring on the painful images in both groups (painful: Mean=69.65, SD=15.99; neutral: Mean=47.76, SD=10.59). The main effect of group (F_1,36_=2.80, P=0.1] and the group×stimuli interaction (F_1,36_=0.09, P=0.76] were not significant. For duration of first fixations, there were no significant main effects for stimuli (F_1,36_=0.43, P=0.51), group (F_1,36_=2.95, P=0.09] and the interaction effect (F_1,36_=2.87, P=0.09). Regarding the total duration of fixations there were no significant main effects for stimuli (F_1,36_=3.20, P=0.08, ηp^2^=0.08), group (F_1,36_=0.614, P=0.439) and the interaction effect (F_1,36_=0.635, P=0.431).

**Figure 3.**
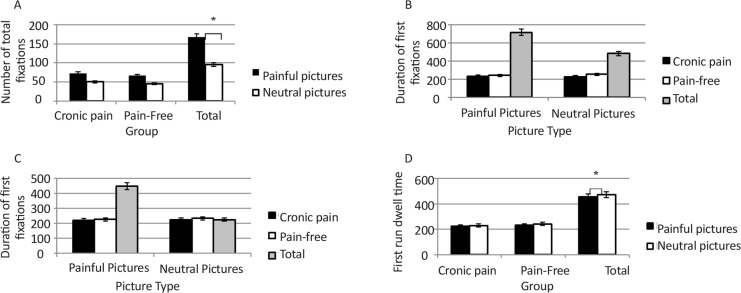
Comparison of indices of sustained attention on neutral and painful pictures between the chronic pain and pain-free groups A mixed model ANOVA showed that most fixations occur on painful images compared to neutral images in both groups (A) and the average first run dwell time for neutral images was generally significantly higher than that of painful images (C)

Regarding the average first run dwell time, there was a significant main effect of picture type (F_1,36_=8.21, P=0.007, ηp^2^=0.18), where average first run dwell time for neutral images was significantly higher than painful images (neutral: Mean=235.43, SD=24.81; painful: Mean=227.12, SD=26.81). However, neither the main effect for group (F_1,36_=1.238, P=0.273] nor the interaction between group×picture type were significant (F_1,36_=1.47, P=0.23) (Figure 3).

## 4. Discussion

The present study aimed to determine whether patients with chronic pain demonstrated attentional biases towards stimuli that depicted painful tasks in comparison to control group, and to characterize the nature of those biases. We predicted that patients with chronic pain would probably fixate first on pain pictures and locate them more quickly than neutral pictures in comparison to the control group, indicating vigilance for pictures of pain-provoking activities. This hypothesis was only partially supported. That is, all participants tended to fixate more often on pictures of pain-provoking activities but for less time than the neutral stimuli. In addition, comparing with patients with chronic pain, control participants were locate neutral pictures earlier than pain-related pictures while for pain patients an opposite pattern of attentional bias was observed. Follow-ups demonstrated that control participants located neutral stimuli more quickly than the pain stimuli, while patients with pain did not show such a bias. This pattern suggests a relative bias for pictures of pain-provoking activities among chronic pain patients in comparison with healthy controls. Interestingly, patients with higher levels of experienced pain were faster in detection of pain-related images.

Regarding the sustained attentional processes, two hypotheses have been proposed in the literature. One hypothesis justifies the existence of the observed attentional bias among patients with chronic pain because of difficulty in disengagement from pain-related stimuli and the other hypothesis considers this bias as the result of subsequent avoidance in attention. Our findings revealed no difference between the chronic pain and control participants in the attentional processes when we take the measures of sustained attention into account. Although patients with pain showed avoidance from pain-provoking stimuli, a similar pattern of bias is observed among healthy controls.

Recent research along with eye tracking studies (Liossi, et al., 2014; Yang, et al., 2013) and prospective studies (Lautenbacher et al., 2011; Lautenbacher et al., 2010; Sharpe, Haggman, Nicholas, Dear, & Refshauge, 2014) have provided evidence supporting the view that the putative process in attentional biases in pain is characterized by vigilance-avoidance (Sharpe, 2014). Interestingly, two previous studies on eye-tracking concluded with contradictory findings when we question individuals’ ability in disengagement from pain-related stimuli. Yang et al. (2013) found that sustained attention was associated with faster disengagement, but Liossi et al. (2014) failed to find such an effect. Our results partially support vigilance-avoidance hypothesis and are consistent with those of Liossi et al. (2014) as in the group of patients with pain, we were unable to identify avoidance. In our study, patients with higher levels of experienced pain fixated more quickly on pain-provoking pictures. One may take this pattern as an argument in support of the assumption behind the vigilance-avoidance hypothesis, though with the current pattern it is still speculative to consider this as a strong evidence.

A meta-analysis of attentional bias studies demonstrated that biases were more evident at longer latencies (e.g. 1250 vs. 500 ms) on dot-probe data (Schoth et al., 2012), indicating that attentional biases in pain can be best characterized by difficulties in disengaging from pain-related stimuli. Consistent with the two previous eye tracking studies (Liossi, et al., 2014; Yang, et al., 2013), we did not find any evidence that patients with chronic pain dwelled longer on pain-related stimuli. Notably, all three studies used different types of stimuli to test this hypothesis. Yang et al. (2013) used word stimuli, Liossi et al. (2014) used face stimuli and we opted for pictures of potentially pain-provoking stimuli. Furthermore, another two eye-tracking studies on healthy pain-free individuals, which targeted fear of pain (Yang, et al., 2012) and catastrophizing (Vervoort, et al., 2013) in their studies, also failed to find evidence of difficulties in disengaging from the pain-related stimuli.

A deeper understanding of methodological limitations (those applies to this study and also to a number of similar studies in the literature) may help us to come up with better design for future studies. Firstly, we only used pairs of neutral-pain pictures and did not include other positive or negative stimuli. Specificity of bias has always been a question in the literature. Liossi et al. (2014) used a visual search task and included other types of emotionally negative stimuli but they failed to find biased toward or away from those stimuli. This remains a question for future studies and its investigation may help us to better understand the underlying mechanism involved in the attentional bias in pain.

Secondly, selection of stimuli in our study and other similar studies is based on evaluation by a group of patients with pain (in our case an independent sample). A number of previous studies provided evidence supporting hypothesis behind the existence of negative interpretation bias among patients with chronic pain or individuals with elevated levels of pain-related concerns (Khatibi, Sharpe, Jafari, Gholami, & Dehghani, 2015; Khatibi et al., 2014). Accordingly, one cannot rule out the possibility that it is the interpretation of the pictures as painful which is responsible for the between group differences rather than the response to pictures of pain-provoking activities, per se.

Thirdly, we opted for a stimulus presentation time of 1000 ms. It is possible that longer exposures to stimuli may help in determining the time course of attention in patients with chronic pain and help understand better patients’ attention for pain-related stimuli. However, Liossi and colleagues (2014) who used a much longer exposure time, encountered problems with fatigue in their participants and hence we opted for a shorter exposure.

In spite of these limitations, the present results have some important theoretical implications for the nature of attentional biases in pain. It has been argued that pain, by its nature, is prioritized in attentional processing for adaptive reasons (Eccleston & Crombez, 1999). Our findings provide evidence in favor of this argument. Both participants with chronic pain and control (despite not rating the pictures as highly likely to cause pain) were initially focused on the pictures depicting potentially painful activities. Further, these pictures received more fixations than neutral pictures, despite the fact that overall less time was spent fixating on the pain-provoking pictures. Hence, the ‘typical’ attentional pattern that appears to be elicited by pictures of activities that could provoke pain appears to be characterized itself by a pattern of vigilance-avoidance.

In conclusion, both healthy individuals and CP patients are more likely to fixate on pictures of pain-provoking pictures. While healthy individuals fixated more quickly on neutral than pain-provoking pictures, chronic pain patients did the reverse, indicating a relative vigilance to pain-provoking pictures. Moreover, the more pain that patients reported, the more quickly they fixated on pain-provoking pictures. However, the experience of pain, itself, did not affect sustained attention. While these results do not provide unqualified support for the vigilance-avoidance hypothesis accounting for attentional biases in pain, they suggest that chronic pain patients do not demonstrate difficulties in disengaging from pain-related stimuli.

## Appendix. Comparison of rating of PHODA images based on VAS between patient and control group using Independent Sample T-test

**Table:**

**Stimulus**	**CP(n=34)**	**Control(n=20)**	**t^*^**

	**M(SD)**	**M(SD)**	
P3	22.35(28.10)	6.45(15.56)	2.67
P5	23.23(28.46)	5.10(14.66)	3.08
P7	34.26(29.33)	11.40(14.38)	3.82
P9	37.41(34.36)	14.80(16.53)	3.25
P10	19.85(23.91)	7.95(11.72)	2.45
P14	50.14(32.90)	17.30(21.62)	4.42
P15	31.17(28.36)	10.60(18.14)	3.24
P17	27.64(26.66)	9.65(12.97)	3.32
P18	32.50(32.94)	13.15(15.21)	2.93
P19	17.55(23.89)	4.45(8.51)	2.90
P22	34.26(29.74)	12.95(17.49)	3.31
P24	11.32(17.72)	2.50(5.23)	2.70
P26	21.61(24.85)	6.25(11.79)	3.06
P31	32.50(31.81)	8.50(13.26)	3.86
P32	25.44(24.66)	8.55(11.89)	3.38

* All P-values≤0.01

## References

[B1] Akbari F. Dehghani M. Khatibi A. Vervoort T 2016 Incorporating Family function into chronic pain disability: The role of catastrophizing. Pain Research and Management, 2016, 1–9. doi: 10.1155/2016/6838596 PMC4904613 27445620

[B2] Andersson G. Haldrup D 2003 Personalized pain words and Stroop interference in chronic pain patients. European Journal of Pain, 7(5), 431–438. doi: 10.1016/s1090-3801(03)00002-8 12935795

[B3] Asghari A. Nicholas M. K 2001 Pain self-efficacy beliefs and pain behaviour. A prospective study. Pain, 94(1), 85–100. doi:10.1016/s0304-3959(01)00344-x 11576748

[B4] Asmundson G. J. Norton P. J. Vlaeyen J. W 2004 Fear-avoidance models of chronic pain: An overview. In Asmundson G. J. Vlaeyen J. W. Crombez G. (Eds.), Understanding and Treating Fear of Pain (pp. 3–24). Oxford: Oxford University Press.

[B5] Asmundson G. J. Vlaeyen J. W. S. Crombez G 2004 Understanding and treating the fear of pain. Oxford: Oxford University Press.

[B6] Asmundson G. J. G. Wright K. D. Hadjistavropoulos H. D. (2005)(2005). Hypervigilance and attentional fixedness in chronic musculoskeletal pain: consistency of findings across modified stroop and dot-probe tasks. The Journal of Pain, 6(8), 497–506. doi: 10.1016/j.jpain.2005.02.012 16084464

[B7] Bayani A. A 2010 Reliability and preliminary evidence of validity of a Farsi version of the depression anxiety stress scales. Perceptual and Motor Skills, 111(1), 107–114. doi: 10.2466/08.13.pms.111.4.107-114 21058592

[B8] Crombez G. Van Ryckeghem D. M. L. Eccleston C. Van Damme S 2013 Attentional bias to pain-related information: A meta-analysis. Pain, 154(4), 497–510. doi: 10.1016/j.pain.2012.11.013 23333054

[B9] Dear B. F. Sharpe L. Nicholas M. K. Refshauge K 2011 Pain-related attentional biases: The importance of the personal relevance and ecological validity of stimuli. The Journal of Pain, 12(6), 625–632. doi: 10.1016/j.jpain.2010.11.010. 21310670

[B10] Dyer A. G. Found B. Rogers D 2006 Visual attention and expertise for forensic signature analysis. Journal of Forensic Sciences, 51(6), 1397–1404. doi: 10.1111/j.1556-4029.2006.00269.x 17199627

[B11] Eccleston C. Crombez G 1999 Pain demands attention: A cognitive–affective model of the interruptive function of pain. Psychological Bulletin, 125(3), 356–366. doi: 10.1037/0033-2909.125.3.356 10349356

[B12] Haggman S. P. Sharpe L. A. Nicholas M. K. Refshauge K. M 2010 Attentional biases toward sensory pain words in acute and chronic pain patients. The Journal of Pain, 11(11), 1136–1145. doi: 10.1016/j.jpain.2010.02.017 20797918

[B13] Khatibi A. Dehghani M. Sharpe L. Asmundson G. J. G. Pouretemad H 2009 Selective attention towards painful faces among chronic pain patients: Evidence from a modified version of the dot-probe. Pain, 142(1), 42–47. doi:10.1016/j.pain.2008.11.020 19201094

[B14] Khatibi A. Sharpe L. Jafari H. Gholami S. Dehghani M 2015 Interpretation biases in chronic pain patients: an incidental learning task. European Journal of Pain, 19(8), 1139–1147. doi: 10.1002/ejp.637 25523038

[B15] Khatibi A. Schrooten M. G. S. Vancleef L. M. G. Vlaeyen J. W. S 2014 An experimental examination of catastrophizing-related interpretation bias for ambiguous facial expressions of pain using an incidental learning task. Frontiers in Psychology, 5, 1002. doi: 10.3389/fpsyg.2014.01002 25278913 PMC4166218

[B16] Lang P. Bradley M. Cuthbert B 2008 International affective picture system (IAPS): Affective ratings of pictures and instruction manual: Technical report A-8. Gainesville: University of Florida.

[B17] Lautenbacher S. Huber C. Baum C. Rossaint R. Hochrein S. Heesen M 2011 Attentional avoidance of negative experiences as predictor of postoperative pain ratings and consumption of analgesics: comparison with other psychological predictors. Pain Medicine, 12(4), 645–653. doi: 10.1111/j.1526-4637.2011.01076.x 21392251

[B18] Lautenbacher S. Huber C. Schöfer D. Kunz M. Parthum A. Weber P. G. 2010 Attentional and emotional mechanisms related to pain as predictors of chronic postoperative pain: a comparison with other psychological and physiological predictors. Pain, 151(3), 722–731. doi: 10.1016/j.pain.2010.08.041 20850220

[B19] Liossi C. Schoth D. E. Godwin H. J. Liversedge S. P 2014 Using eye movements to investigate selective attention in chronic daily headache. Pain, 155(3), 503–510. doi: 10.1016/j.pain.2013.11.014 24287436

[B20] Lovibond S. Lovibond P. F 1995 Manual for the depression anxiety stress scales. Sydney: Psychology Foundation of Australia.

[B21] Mogg K. Millar N. Bradley B. P 2000 Biases in eye movements to threatening facial expressions in generalized anxiety disorder and depressive disorder. Journal of Abnormal Psychology, 109(4), 695–704. doi: 10.1037/0021-843x.109.4.695 11195993

[B22] Mohammadi S. Dehghani M. Sharpe L. Heidari M. Sedaghat M. Khatibi A 2012 Do main caregivers selectively attend to pain-related stimuli in the same way that patients do? Pain, 153(1), 62–67. doi: 10.1016/j.pain.2011.08.021 22001657

[B23] Mohammadi S. Dehghani M. Khatibi A. Sanderman R. Hagedorn M 2015 Caregivers’ attentional bias to pain: does it affect caregiver accuracy in detecting patient pain behaviors? Pain, 156(1), 123–130. doi: 10.1016/j.pain.0000000000000015 25599308

[B24] Roelofs J. McCracken L. Peters M. L. Crombez G. Van Breukelen G. Vlaeyen J. W 2004 Psychometric evaluation of the Pain Anxiety Symptoms Scale (PASS) in chronic pain patients. Journal of Behavioral Medicine, 27(2), 167–183. doi: 10.1023/b:jobm.0000019850.51400.a6 15171105

[B25] Roelofs J. Peters M. L. McCracken L. Vlaeyen J. W. 2003 The Pain Vigilance and Awareness Questionnaire (PVAQ): further psychometric evaluation in fibromyalgia and other chronic pain syndromes. Pain, 101(3), 299–306. doi: 10.1016/s0304-3959(02)00338-x 12583873

[B26] Roelofs J. Peters M. L. Zeegers M. P. A. Vlaeyen J. W. S 2002 The modified Stroop paradigm as a measure of selective attention towards pain-related stimuli among chronic pain patients: A meta-analysis. European Journal of Pain, 6(4), 273–281. doi: 10.1053/eujp.2002.0337 12161093

[B27] Roland M. Fairbank J 2000 The Roland–Morris disability questionnaire and the Oswestry disability questionnaire. Spine, 25(24), 3115–3124. doi: 10.1097/00007632-200012150-00006 11124727

[B28] Schoth D. E. Nunes V. D. Liossi C 2012 Attentional bias towards pain-related information in chronic pain; a meta-analysis of visual-probe investigations. Clinical Psychology Review, 32(1), 13–25. doi: 10.1016/j.cpr.2011.09.004 22100743

[B29] Sharpe L 2014 Attentional biases in pain: More complex than originally thought? Pain, 155(3), 439–440. doi: 10.1016/j.pain.2013.12.020 24342463

[B30] Sharpe L. Haggman S. Nicholas M. Dear B. F. Refshauge K 2014 Avoidance of affective pain stimuli predicts chronicity in patients with acute low back pain. Pain, 155(1), 45–52. doi: 10.1016/j.pain.2013.09.004 24028848

[B31] Swinkels-Meewisse E. Swinkels R. Verbeek A. Vlaeyen J. Oostendorp R 2003 Psychometric properties of the Tampa Scale for kinesiophobia and the fear-avoidance beliefs questionnaire in acute low back pain. Manual Therapy, 8(1), 29–36. doi: 10.1054/math.2002.0484. 12586559

[B32] Van Damme S. Crombez G. Eccleston C 2004 The anticipation of pain modulates spatial attention: Evidence for pain-specificity in high-pain catastrophizers. Pain, 111(3), 392–399. doi: 10.1016/j.pain.2004.07.022 15363884

[B33] Van Ryckeghem D. M. Crombez G. Goubert L. De Houwer J. Onraedt T. Van Damme S 2013 The predictive value of attentional bias towards pain-related information in chronic pain patients: A diary study. Pain, 154(3), 468–475. doi: 10.1016/j.pain.2012.12.008 23375161

[B34] Vervoort T. Trost Z. Prkachin K. M. Mueller S. C 2013 Attentional processing of other’s facial display of pain: An eye tracking study. Pain, 154(6), 836–844. doi: 10.1016/j.pain.2013.02.017 23561271

[B35] Vlaeyen J. W. S. Linton S. J 2012 Fear-avoidance model of chronic musculoskeletal pain: 12 years on. Pain, 153(6), 1144–1147. doi: 10.1016/j.pain.2011.12.009 22321917

[B36] Yang Z. Jackson T. Chen H 2013 Effects of chronic pain and pain-related fear on orienting and maintenance of attention: An eye movement study. The Journal of Pain, 14(10), 1148–1157. doi: 10.1016/j.jpain.2013.04.017 23850178

[B37] Yang Z. Jackson T. Gao X. Chen H 2012 Identifying selective visual attention biases related to fear of pain by tracking eye movements within a dot-probe paradigm. Pain, 153(8), 1742–1748. doi: 10.1016/j.pain.2012.05.011 22717101

